# Symptom Perceptions in Functional Disorders, Major Health Conditions, and Healthy Controls: A General Population Study

**DOI:** 10.32872/cpe.7739

**Published:** 2022-12-22

**Authors:** Angelika Weigel, Thomas Meinertz Dantoft, Torben Jørgensen, Tina Carstensen, Bernd Löwe, John Weinman, Lisbeth Frostholm

**Affiliations:** 1 Department of Psychosomatic Medicine and Psychotherapy, University Medical Center Hamburg-Eppendorf, Hamburg, Germany; 2 The Research Clinic for Functional Disorders and Psychosomatics, Aarhus University Hospital, Aarhus, Denmark; 3 Center for Clinical Research and Prevention, Bispebjerg and Frederiksberg Hospital, Capital Region of Denmark, Denmark; 4 Department of Public Health, Faculty of Medical Sciences, University of Copenhagen, Copenhagen, Denmark; 5 Faculty of Medicine, Aalborg University, Aalborg, Denmark; 6 Department of Clinical Medicine, Aarhus University, Aarhus, Denmark; 7 School of Cancer & Pharmaceutical Sciences, King's College London, London, United Kingdom; Philipps-University of Marburg, Marburg, Germany

**Keywords:** symptom perceptions, functional disorders, epidemiological study, quality of life, common-sense model of illness, personality traits

## Abstract

**Background:**

The present study investigated differences in symptom perceptions between individuals with functional disorders (FD), major health conditions, and FDs + major health conditions, respectively, and a group of healthy individuals. Furthermore, it investigated the relevance of FDs among other health-related and psychological correlates of symptom perceptions in the framework of the Common Sense Model of Self-Regulation (CMS).

**Method:**

This cross-sectional study used epidemiological data from the Danish Study of Functional Disorders part two (N = 7,459 participants, 54% female, 51.99 ± 13.4 years). Symptom perceptions were assessed using the Brief Illness Perception Questionnaire (B-IPQ) and compared between the four health condition groups. Multiple regression analyses were performed to examine associations between symptom perceptions, FDs, and other health-related and psychological correlates from the CMS framework.

**Results:**

Individuals with FDs (n = 976) and those with FDs + major health conditions (n = 162) reported less favorable symptom perceptions compared to the other two groups, particularly regarding perceived consequences, timeline, and emotional representations (effect size range Cohen’s d = 0.12-0.66). The presence of a FD was significantly associated with all B-IPQ items, even in the context of 16 other relevant health-related and psychological correlates from the CMS framework, whereas symptom presence last year or last week was not.

**Conclusion:**

In the general population, symptom perceptions seem to play a more salient role in FD than in individuals with well-defined physical illness. Symptom perceptions should therefore be targeted in both primary and secondary interventions for FDs.

## Highlights

- Symptom perceptions were poorest in individuals with functional disorders with and without co-occuring major health conditions.
- Functional disorders in oneself and in the family were associated with symptom perceptions.
- Symptom presence last year or last week was not associated with symptom perceptions.

Experiencing physical symptoms is a common everyday phenomenon in the general population (Hinz et al., 2017). Their perception and appraisal are results of multidimensional processes that go beyond a recognition of peripheral bodily changes (Petersen et al., 2011). In major health conditions (e.g., cancer, heart attack), the relationship between peripheral bodily dysfunctions and self-reported symptoms is weaker in chronic multisymptomatic than in acute monosymptomatic diseases (Janssens et al., 2011). In functional disorders, i.e., bothersome physical conditions that are not better explained by physical diseases or mental disorders and are associated with reduced health-related quality of life, evidence suggests a weaker relation between physical parameters (e.g., respiratory changes after gradually increased ventilation) and symptom perceptions (e.g., perceived dyspnea) compared to healthy controls (Bogaerts et al., 2010). These varying associations between peripheral bodily changes and symptom perceptions underline the relevance of cognitive and emotional processes in symptom perception and appraisal (Van den Bergh et al., 2017).

Symptom perceptions describe dynamic mental representations and personal ideas that individuals generate to make sense of and respond to their symptoms (Broadbent et al., 2015). Among numerous empirically tested theoretical models of symptom perception and appraisal (Whitaker et al., 2015), the Common-Sense Model of Self Regulation is particularly established (CSM; Leventhal et al., 2016). According to the CSM, individuals’ mental models of experienced symptoms include cognitive representations of the symptom identity (lay diagnosis), the coherence and the perceived timeline, the control over and consequences of the experienced symptoms as well as emotional representations of symptom concerns and emotional reactions. Symptom perceptions thereby directly affect the coping efforts that may be more or less beneficial. Individuals then appraise the effects of these coping efforts, which may result in changes to their cognitive representations and emotional responses in a feedback loop. However, while healthy individuals can form their symptom perceptions based on their experience that symptoms are usually non-threatening and short-lived everyday phenomena and individuals with chronic diseases usually receive a biomedical explanation of their symptoms and a diagnostic label with an associated treatment rational, individuals with functional disorders lack these aspects. Instead, individuals with functional disorders are often confronted with inconclusive medical findings and receive no diagnostic label or external information about the possible course of the disease, which might negatively influence their symptom perceptions.

Symptom perceptions have an impact on health outcomes in both mental and somatic disorders (Dempster et al., 2015; Hagger et al., 2017). For example, one methodologically rigorous study that investigated illness perceptions in a primary healthcare sample with diverse new health complaints provided evidence for the impact of symptom perceptions on quality of life (Frostholm et al., 2007). Furthermore, there is a large body of literature on the influence of symptom perceptions in clearly defined medical conditions on various health outcomes (Aalto et al., 2006; De Gucht, 2015; O’Donovan et al., 2016; Tiemensma et al., 2016; Timmers et al., 2008; Tribbick et al., 2017; van Erp et al., 2017; Xiong et al., 2018). Despite valuable insights into the relevance of symptom perceptions on health outcomes, previous studies have rarely investigated symptom perceptions in individuals with functional disorders with potential co-occuring medical conditions. Research into this area is crucial as suggested by a Dutch epidemiological study showing that the functional impairments associated with functional disorders are similar in severity to those in major health conditions (Joustra et al., 2015). In addition, more negative symptom perceptions have been observed in individuals with functional gastrointestinal disorders compared with patients with peptic ulcer or reflux esophagitis (Xiong et al., 2018) and functional disorders might co-occur with medical conditions (Halpin & Ford, 2012).

According to the CSM, a number of contextual, health-related, and psychological factors may influence the formation of symptom perceptions. A recent systematic review on so-called modifiable correlates of symptom perceptions observed an association between higher symptom severity and less favorable symptom perceptions in different somatic conditions (Arat et al., 2018). The same review highlighted a negative influence of depression and anxiety on symptom perceptions, with the limitation that no differentiation was made between lifetime mental disorders and the current presence of symptoms. Only few studies have considered other mental comorbidities than depression and anxiety. Two studies investigated the influence of post-traumatic stress disorder (PTSD) on symptom perceptions in patients with a myocardial infarction and observed significantly less favorable symptom perceptions in patients with PTSD symptomatology compared with those without (Princip et al., 2018; Sheldrick et al., 2006). In contrast, many studies have investigated coping and symptom perceptions. A meta-analysis by Dempster and colleagues concluded that symptom perceptions and coping explain a valuable amount of variance in distress outcomes across a range of physical health conditions (Dempster et al., 2015).

One cross-sectional study investigated the association between Type D personality and illness perceptions in colorectal cancer survivors and observed significantly less favorable symptom perceptions in those with high Type D personality traits (Mols et al., 2012). However, the concept of Type D personality has been criticized in favor of the Big Five personality traits (neuroticism, extraversion, openness, agreeableness, conscientiousness; Horwood & Anglim, 2017). Furthermore, there is evidence that personality traits are more relevant to symptom perceptions than current illness severity (Goetzmann et al., 2005), and that symptom perceptions at least partially mediate the association between personality traits and coping (Rassart et al., 2014). Within this body of literature on correlates of symptom perceptions in the framework of the CMS, the possible influence of functional disorders in a patient or his/her significant others has not yet been investigated.

Knowledge of symptom perceptions within the CSM framework from a large representative general population sample can help shed light on the possible differences in symptom perceptions in functional disorders and somatic diseases, respectively. Such an investigation would increase the evidence base for the current theoretical understanding of the role of specific symptom perceptions in functional disorders. Furthermore, it may pave the way for the identification of intervention components to improve symptom management and improve health outcomes as has been shown in patients with myocardial infarction (Petrie et al., 2002) and severe functional disorders (Christensen et al., 2015).

The first aim of the present epidemiological study was to compare symptom perceptions in healthy individuals and individuals with either functional disorders, major health conditions or both. We hypothesized that there would be differences between the four health condition groups, with particularly less favorable symptom perceptions in individuals with functional disorders. The second aim was to examine whether the presence of a functional disorder in a participant or his/her significant others would explain meaningful variance in symptom perceptions besides a large number of other possible correlates of symptom perceptions from the CMS framework by means of an exploratory approach.

## Method

### Study Population

Data collection took place in the context of the “Danish study of Functional Disorders” (DanFunD; Dantoft et al., 2017). The complete DanFunD sample comprises a random sample of 9,656 participants aged between 18-76 years from the Danish general population living in the western part of greater Copenhagen (participation rate 33.7%). Recruitment occurred in two cross-sectional waves with the same eligibility criteria: DanFunD part one from 2011 to 2012 (2,308 participants) and DanFunD part two from 2012 to 2015 (7,493 participants). All DanFunD participants completed a general health examination and a self-report questionnaire battery at the Research Centre for Prevention and Health, Glostrup, Denmark. The DanFunD part two self-report questionnaire battery included a questionnaire on symptom perceptions, and this cohort was therefore eligible for the present study. All participants gave their written informed consent prior to study participation. The study was approved by the Ethical Committee of Copenhagen Country (KA-2006-0011, H-3-2011-081, H-3-2012).

### Measures

#### Symptom Perceptions

The Danish version of the B-IPQ was applied to assess symptom perceptions with eight numerous rating scales (range 1–10, for item wording see Table 2, Broadbent et al., 2006). The B-IPQ uses a single-item scaling to measure symptom perceptions based on the CSM with five items related to cognitive perceptions, two items to emotional aspects and one item to the understanding of an illness. Participants were instructed only to fill out the B-IPQ items if they had experienced symptoms during the last year according to the BDS checklist (see below) or the last week (SCL-90 somatization subscale). As symptom perceptions were assessed with respect to physical symptoms and not to a certain illness, the B-IPQ item assessing illness *identity* was removed. Items assessing personal control, treatment control and coherence were reversed to facilitate interpretation, i.e., that higher scores indicate less control and less coherence.

#### Four Health Condition Groups

The questionnaire set comprised a predefined 22-item list that covered diagnosed major health conditions, functional disorders and mental health disorders that were categorically answered (yes/no) to the question “Has a doctor ever told you that you have/had…”. Participants were asked to answer this 22-item list with regard to themselves and each family member (i.e., fathers, mothers, siblings). Within this list, cancer, heart attack and thrombosis or embolism in the brain were operationalized as major health conditions. Fibromyalgia, chronic fatigue, irritable bowel syndrome, whiplash syndrome, and multiple chemical sensitivity were operationalized as functional disorders. Lifetime depression and anxiety were operationalized as mental disorders. Of note, the list did not include questions on mental disorders in the family. In each case, a major health condition, functional disorder, or mental disorder was evaluated as being present either in the patient or in the family if one of the respective items was answered positively. The four health condition groups were: functional disorders, major health conditions, functional disorders and major health conditions, and healthy (i.e., no major health condition or functional disorder).

#### Perceived Symptoms

The Bodily Distress Syndrome (BDS) checklist (Budtz-Lilly et al., 2015) uses a Likert-scale to assess 25 symptoms related to the cardiopulmonary, gastrointestinal, musculoskaletal and general symptom clusters of the diagnostic concept of the Bodily Distress Syndrome. The Danish version of the BDS checklist was applied to assess the presence of physical symptoms *during the last year*. As we focussed on the number of symptoms during the last year rather than the burden of each symptom, answers were dichotomized (0 = *not at all*; 1 = *little to a lot*) and summed up with higher values indicating a higher number of symptoms (range 0-32). Likewise, physical symptoms *during the last week* were operationalized through the 12-item sum score of the SCL-90 somatization subscale (range 0-12, Cronbach’s alpha in this sample = 0.80; Olsen et al., 2004).

#### Psychological Factors

Current symptoms of depression and anxiety were assessed using the 8-item sum score of the SCL-90 mental distress subscale (range 0-24, Cronbach’s alpha in this sample = 0.87; Fink et al., 2004).

Personality traits were operationalized based on the NEO-Five Factor Inventory that assesses the personality traits neuroticism, extraversion, agreeableness, openness, and conscientiousness through 60 Likert-scaled items (subscale range 0-48 points; Körner et al., 2002).

The number of adverse life events was operationalized through the Cumulative Lifetime Adversity Measure (range 0-37, additional item to mention specific life adversaries). The questionnaire asks respondents whether they ever experienced one or more of 37 different negative life events (Carstensen et al., 2020).

The 10-item Perceived Stress Scale with a Likert-scaled answering format was used to assess current stress (sum score range 0-40 points, Cronbach’s alpha in this sample = 0.87; Cohen et al., 1983).

The 10-item General Self-Efficacy Scale with a Likert-scaled answering format was applied to assess coping abilities (sum score range 0-30 points, Cronbach’s alpha in this sample = 0.91; Luszczynska et al., 2005).

#### Self-Perceived Health

One Likert-scaled item of the 12-item Short Form Health Survey (Ware et al., 1996) was applied to assess self-perceived health as an indicator of health related quality of life.

#### Objective Health Measures

Body mass index (BMI = kg/m^2^) and waist-to-hip ratio were obtained.

#### Sociodemographic Aspects

Age, sex and years of school education (≤10 years = “elementary school education” >10 years = “beyond elementary school education”) were included.

### Statistical Analyses

Participants with a minimum of four answered B-IPQ items (i.e., completers) and those with zero to three answered items were compared with regard to sex, age, marital status, and school education to identify potential selection biases. The four health condition groups were compared with regard to sociodemographic and clinical characteristics using χ^2^-tests for categorical (sex, marital status, school education) and ANOVA for metric variables (age, BMI, waist-to-hip ratio, self-perceived health).

First study aim: B-IPQ items were compared between each of the four health condition groups applying an ANCOVA with age and sex as covariates and Bonferroni corrected post hoc tests. Adjusted means, standard errors (*SE*) and in case of significant differences effect sizes (Cohen’s *d*) are reported.

Second study aim: Seven multiple regression analyses with each including a total of 18 independent variables were applied to examine associations between the B-IPQ items and functional disorders (own; in the family) as well as other health-related (own major health condition or in the family, symptom presence in the last year and the last week) and psychological correlates of symptom perceptions (own mental disorder, mental distress, perceived stress, coping ability, number of adverse life events, personality traits) and sociodemographic variables (i.e., sex, age,) in the framework of the CMS. B-IPQ items were log10 transformed due to skewness and linearity.

No imputation procedure was applied on the study variables and the maximum available information was used in each analysis. IBM SPSS version 25 (SPSS Inc., Chicago, IL, USA) was used for all analyses. The significance level was set at *p* < .05 with adjustments in case of multiple testing.

## Results

Among the 7,459 participants, 7% affirmed on the predefined list that a doctor told them they had cancer, 2% a heart attack and 2% thrombosis or embolism in the brain. Further 1% affirmed to have been told to have fibromyalgia, 1% chronic fatigue, 12% irritable bowel syndrome, 3% whiplash syndrome, and 2% multiple chemical sensitivity. Sociodemographic and clinical characteristics differed significantly between healthy individuals and the other three health condition groups with regard to age, sex, marital status, BMI, and waist-to-hip ratio (see Table 1). Within this total sample, 2,135 did not answer any B-IPQ items (84% healthy individuals, 9% major health conditions, 6% functional disorders, 1% both). An additional 107 answered one to three (76%, 10%, 6%, 3%,) and 5,217 participants answered ≥4 B-IPQ items (71% of the cohort).

**Table 1:** Sample Characteristics of Participants From the DanFunD Part Two Study Sample

Variable	Healthy *n* = 5524	Major Health Condition *n* = 601	Functional Disorder *n* = 976	Major Health Condition + Functional Disorder *n* = 162	Statistics
**Sex** % (*n*) female	51 (2821)	52 (311)	69 (672)	67 (108)	χ^2^ = 117.377, *p* < .001
**Age** *M* (*SD*)	50.49 (13.50)	59.94 (9.19)	53.29 (12.68)	60.21 (8.37)	*F* = 125.064, *p* < .001
**Marital status** % (*n*) married	64 (3544)	72 (429)	66 (639)	67 (109)	χ^2^ = 95.259, *p* < .001
**School education** % (*n*) > 10 years	56 (2972)	52 (303)	53 (496)	50 (80)	χ^2^ = 9.103, *p* = .028
**Body Mass Index** *M* (*SD*)	25.84 (4.53)	27.15 (4.57)	26.30 (5.06)	27.08 (4.60)	*F* = 18.726, *p* < .001
**Waist-to-Hip Ratio** *M* (*SD*)	0.88 (0.09)	0.91 (0.10)	0.87 (0.09)	0.90 (0.09)	*F* = 22.888, *p* < .001
**Self-perceived health**^a^ *M* (*SD*)	2.39 (0.76)	2.76 (0.80)	2.86 (0.83)	3.17 (0.79)	*F* = 166.024, *p* < .001

*Note.* *M* = Mean; *SE* = standard deviation; Cancer, heart attack and thrombosis or embolism in the brain were operationalized as major health conditions from a predefined list of 22 diseases; Fibromyalgia, chronic fatigue, irritable bowel syndrome, whiplash syndrome, and multiple chemical sensitivity were operationalized as functional disorders from the same list of diseases.

^a^Increasing scores equal a worse self-perceived health.

### Aim 1: Comparison of Symptom Perceptions in the Four Health Condition Groups

**Figure 1:**
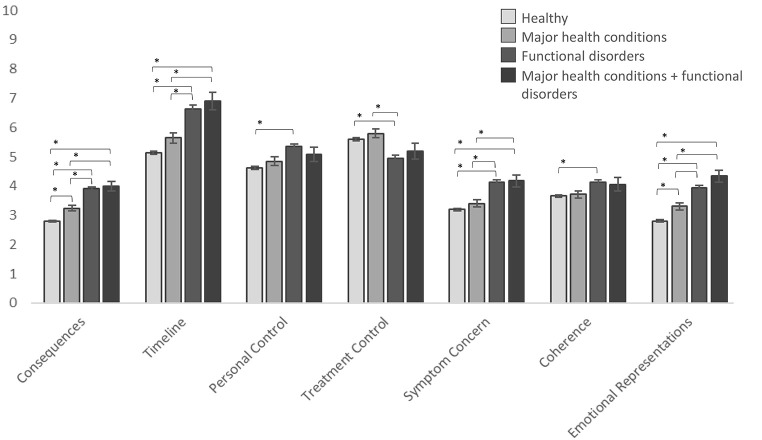
Mean Comparisons of Symptom Perceptions as Assessed With the B-IPQ in the Four Health Condition Groups Adjusted for Age and Sex *Note*. x-Axis = Items of the Brief Illness Perception Questionnaire (B-IPQ), y-axis = Visual Analog Scale, range of 0-10. * = significant group difference. Error bars represent Standard Errors. Cancer, heart attack, and thrombosis or embolism in the brain were operationalized as major health conditions from a predefined list of 22 diseases; fibromyalgia, chronic fatigue, irritable bowel syndrome, whiplash syndrome, and multiple chemical sensitivity were operationalized as functional disorders from the same list of diseases.

All health condition groups differed significantly from each other with regard to the B-IPQ subscale items when controlling for age and sex (see Figure 1, Table 2 and Appendix).

Participants with **major health conditions** reported significantly less favorable *consequences* (Cohen’s *d* = 0.20) and *emotional representations* (Cohen’s *d* = 0.17) than healthy participants. Participants with major health conditions also reported significantly more favorable *consequences* (Cohen’s *d* = 0.32), *timeline* (Cohen’s *d* = 0.27), *symptom concern* (Cohen’s *d* = 0.31), and *emotional representations* (Cohen’s *d* = 0.45) as well as significantly less favorable *treatment control* (Cohen’s *d* = 0.27) than participants with functional disorders. With the exception of *treatment control*, a similar picture occurred between participants with major health conditions and those with functional disorders and major health conditions (Cohen’s *d* range = 0.30–0.37).

**Table 2:** Symptom Perceptions as Assessed With the B-IPQ in Four Health Condition Groups Adjusted for Age and Sex

B-IPQ item	Healthy *n* = 5524	Major Health Conditions *n* = 601	Functional Disorders *n* = 976	Major Health Condition + Functional Disorders *n* = 162	Statistics
**Consequences** *M* (*SE*) *How much do your symptoms affect your life?*	2.79 (0.03)	3.24 (0.10)	3.90 (0.07)	3.99 (0.17)	*F* = 77.670, *df* = 3, *p* < .001
**Timeline** *M* (*SE*) *How long do you think your symptoms will last?*	5.13 (0.06)	5.64 (0.17)	6.64 (0.12)	6.89 (0.29)	*F* = 50.959, *df* = 3, *p* < .001
**Personal Control^a^** *M* (*SE*) *How much control do you feel you have over your symptoms?*	4.62 (0.05)	4.84 (0.15)	5.34 (0.10)	5.08 (0.25)	*F* = 13.872, *df* = 3, *p* < .001
**Treatment Control^a^** *M* (*SE*) *How much do you think your treatment can help your symptoms?*	5.50 (0.05)	5.80 (0.16)	4.95 (0.11)	5.18 (0.27)	*F* = 10.959, *df* = 3, *p* < .001
**Symptom Concern** *M* (*SE*) *How concerned are you about your symptoms?*	3.20 (0.04)	3.40 (0.12)	4.13 (0.08)	4.18 (0.20)	*F* = 40.059, *df* = 3, *p* < .001
**Coherence^a^** *M* (*SE*) *How well do you feel you understand your symptoms?*	3.66 (0.04)	3.71 (0.13)	4.13 (0.09)	4.06 (0.23)	*F* = 7.687, *df* = 3, *p* < .001
**Emotional Representations** *M* (*SE*) *How much do your symptoms affect your emotionally? (e.g. make you angry, scared, upset or depressed)*	2.81 (0.04)	3.31 (0.12)	3.95 (0.08)	4.34 (0.20)	*F* = 69.459, *df* = 3, *p* < .001

*Note*. B-IPQ = Brief Illness Perception Questionnaire; item wordings are in italics. *M* = Mean; *SE* = standard error. Cancer, heart attack and thrombosis or embolism in the brain were operationalized as major health conditions from a predefined list of 22 diseases; Fibromyalgia, chronic fatigue, irritable bowel syndrome, whiplash syndrome, and multiple chemical sensitivity were operationalized as functional disorders from the same list of diseases.

^a^Reversed item, age groups comprise missing values.

Participants with **functional disorders** reported significantly less favorable symptom perceptions than healthy individuals on all but one B-IPQ subscales (Cohen’s *d* range = 0.16–0.56), i.e., treatment control was significantly more favorable in participants with functional disorders. Participants with **functional disorders and major health conditions** reported significantly less favorable *consequences* (Cohen’s *d* = 0.32), *timeline* (Cohen’s *d* = 0.58), *symptom concern* (Cohen’s *d* = 0.42) and *emotional representations* (Cohen’s *d* = 0.66) compared to healthy participants. Notably, participants with functional disorders and those with both major health conditions and functional disorders reported comparable B-IPQ subscale item scores.

### Aim 2: Correlation Between Functional Disorders in Oneself and Significant Others and Symptom Perceptions in the Context of Other Possible Correlates From the CMS Framework

There was no evidence of multi-collinearity as assessed by tolerance values greater than 0.1 and VIF between 1.056 and 3.298. There was indepence of residuals as indicated by Durbin-Watson values between 1.958 and 2.041. The assumption of normality was met as assessed by Q-Q Plots.

Higher, i.e., more negative, perceived *consequences* were significantly associated with own and family functional disorders, own major health conditions, mental disorders, higher mental distress and perceived stress, and more adverse life events (for regression coefficients, standard errors, 95% confidence intervals and model summary, see Table 3).

Higher, i.e., more negative, perceived *timeline* was significantly associated with own and family functional disorders, own major health conditions, higher levels of mental distress, more adverse life events, and lower levels of extraversion.

Higher, i.e. less, perceived *personal control* was significantly associated with own functional disorders, higher levels of mental distress, and perceived stress as well as a lower coping ability, lower levels of conscientiousness, and female sex.

Higher, i.e. less, perceived *treatment control* was significantly associated with, own functional disorders, the absence of functional disorders in the family, lower levels of extraversion and agreeableness, and younger age.

Higher, i.e. more negative, perceived *symptom concerns* were significantly associated with own and family functional disorders, higher mental distress and perceived stress and female sex.

Higher, i.e. less, *coherence* was significantly associated with own functional disorders, the absence of a mental disorder, higher levels of mental distress and perceived stress as well as a lower coping ability, higher levels of neuroticisms and lower levels of openness and agreeableness, younger age and female sex.

Higher, i.e. more negative, *emotional representations* were significantly associated with own and family functional disorders and major health conditions, mental disorders and higher levels mental distress, perceived stress, and neuroticism.

**Table 3:** Summary of Multiple Regression Analyses to Predict Symptom Perceptions in a Danish Population-Based Sample

Variable	Consequences			Timeline			Symptom control			Treatment control		
	*B*	*SE*	95% CI	*B*	*SE*	95% CI	*B*	*SE*	95% CI	*B*	*SE*	95% CI
Functional Disorders and major health conditions												
Own functional disorders	1.26**	1.02	[1.15, 1,32]	1.34**	1.03	[1.25, 1.43]	1.14**	1.03	[1.08, 1.21]	1.06**	1.19	[-0.83, -0.95]
Functional disorders in family	1.05**	1.02	[1.20, 1.10]	1.06*	1.03	[1.00, 1.12]	1.04	1.03	[-0.99, 1.09]	-0.89*	1.03	[-0.89, -0.99]
Own major health conditions	1.07*	1.03	[1.01, 1.14]	1.09*	1.04	[1.00, 1.19]	0.99	1.04	[-0.92, 1.06]	-0.94	1.03	[-0.96, 1.12]
Major health conditions in family	1.02	1.03	[-1.01, 1.07]	1.05	1.04	[0.98, 1.13]	1.00	1.03	[-0.94, 1.07]	-1.04	1.04	[-0.92, 1.06]
Symptoms last year	1.00	1.00	[-0.97, 1.00]	1.00	1.00	[-0.99, 1.00]	-1.00	1.00	[-1.00, 1.00]	-0.99	1.04	[-1.00, 1.01]
Symptoms last week	1.00	1.00	[-0.99, 1.10]	-1.00	1.01	[-0.99, 1.01]	-1.00	1.01	[-0.99, 1.01]	-1.00	1.00	[-0.99, 1.01]
Psychological correlates of symptom perceptions												
Mental disorders	1.06*	1.03	[0.99, 1.12]	-1.04	1.04	[-0.96, 1.12]	-0.94	1.03	[-0.88, 1.00]	-1.00	1.01	[-0.88, 1.02]
Mental distress	1.03**	1.00	[1.00, 1.03]	1.02**	1.00	[-1.01, 1.03]	1.02**	1.00	[1.01, 1.02]	0.95	1.04	[-0.99, 0.00]
Perceived stress	1.01**	1.00	[1.02, 1.02]	1.00	1.00	[-1.00, 1.01]	1.02**	1.00	[1.01, 1.02]	1.00	1.00	[-0.99, 1.01]
Coping ability	1.00	1.00	[-1.01, 1.01]	1.00	1.00	[-1.00, 1.01]	-0.99**	1.00	[-0.99, -1.00]	-1.00	1.00	[-0.99, 1.00]
Adverse life events	1.01**	1.00	[1.00, 1.02]	1.02**	1.00	[1.01, 1.03]	1.01	1.00	[-1.00, 1.02]	-0.99	1.00	[-0.99, 1.00]
Neuroticism	1.00	1.00	[-1.01, 1.01]	1.00	1.00	[-1.00, 1.01]	1.00	1.00	[1.00, 1.01]	-0.99	1.00	[-0.99, 1.00]
Extraversion	1.00	1.00	[-1.00, 1.00]	-0.99**	1.00	[-0.99, -1.00]	-1.00	1.00	[-0.99, 1.00]	-0.99**	1.00	[-0.98, -0.99]
Openness	1.00	1.00	[-1.00, 1.00]	-1.00	1.00	[-0.99, 1.00]	-1.00	1.00	[-0.99, 1.00]	-0.99	1.00	[-1.00, 1.01]
Agreeableness	1.00	1.00	[-0.99, 1.01]	1.00	1.00	[-1.00, 1.01]	-1.00	1.00	[-0.99, 1.00]	-1.00**	1.00	[-0.99, -1.00]
Conscientiousness	1.00	1.00	[-1.00, 1.00]	-1.00	1.00	[-0.99, 1.01]	-0.99**	1.00	[-0.99, -1.00]	-0.99	1.00	[-0.99, 1.01]
Sociodemographic factors												
Sex	1.02	1.02	[-0.99, 1.07]	-0.98	1.03	[-0.92, 1.03]	-0.95*	1.03	[-0.91, -1.00]	-1.00	1.00	[-0.98, 1.09]
Age	1.00	1.00	[-0.98, 1.00]	1.00	1.00	[-1.00, 1.00]	-1.00	1.00	[-1.00, 1.00]	-1.03**	1.03	[-0.99, -1.00]
Model summary	*F*_18,4038_ = 42.228, *p* = < .001 adj. *R*^2^ = 0.155 Durbin-Watson = 1.988 VIF_max_ = 3.290 (Neuroticism)			*F*_18,3965_ = 17.377, *p* = < .001 adj. *R*^2^ = .069 Durbin-Watson = 1.967 VIF_max_ = 3.298 (Neuroticism)			*F*_18, 3986_= 26.894, *p* = < .001 adj. *R*^2^ = .104 Durbin-Watson = 2.041 VIF_max_ = 3.295 (Neuroticism)			*F*_18, 3971_= 5.375, *p* = < .001 adj. *R*^2^ = .019 Durbin-Watson = 1.958 VIF_max_ = 3.277 (Neuroticism)		

**Table 3 [continued]:** Summary of Multiple Regression Analyses to Predict Symptom Perceptions in a Danish Population-Based Sample

Variable	Symptom concerns			Coherence			Emotional representations		
	*B*	*SE*	95% CI	*B*	*SE*	95% CI	*B*	*SE*	95% CI
Functional Disorders and major health conditions									
Own functional disorders	0.08**	1.03	[1.14, 1.27]	1.13**	1.03	[1.07, 1.20]	1.04**	1.15	[1.17, 1.29]
Functional disorders in family	1.06*	1.02	[1.01, 1.11]	1.02	1.03	[-0.98, 1.08]	1.23**	1.03	[1.02, 1.12]
Own major health conditions	1.02	1.04	[0.95, 1.10]	-0.97	1.04	[-0.90, 1.05]	1.07**	1.02	[1.04, 1.18]
Major health conditions in family	1.03	1.03	[-0.98, 1.10]	1.03	1.03	[-0.96, 1.09]	1.11	1.03	[-0.95, 1.06]
Symptoms last year	1.00	1.00	[-1.00, 1.01]	-1.00	1.00	[-0.99, 1.00]	1.01	1.03	[-1.00, 1.00]
Symptoms last week	1.00	1.00	[-0.99, 1.01]	1.00	1.01	[-0.99, 1.02]	1.00	1.00	[-0.99, 1.01]
Psychological correlates of symptom perceptions									
Mental disorders	-0.98	1.03	[-0.92, 1.04]	-0.91*	1.03	[-0.86, -0.98]	1.00**	1.00	[1.03, 1.15]
Mental distress	1.04**	1.00	[1.03, 1.05]	1.01**	1.00	[1.01, 1.02]	1.09**	1.03	[1.04, 1.06]
Perceived stress	1.01**	1.00	[1.01, 1.02]	1.01**	1.00	[1.00, 1.02]	1.05**	1.00	[1.01, 1.02]
Coping ability	1.00	1.00	[-0.99, 1.00]	-0.99**	1.00	[-0.98, -0.99]	-1.02	1.00	[-0.99, 1.00]
Adverse life events	1.01	1.00	[-1.00, 1.01]	-0.99	1.00	[-0.99, 1.00]	1.00	1.00	[-1.00, 1.01]
Neuroticism	1.00	1.00	[1.00, 1.01]	1.01*	1.00	[1.00, 1.01]	1.00**	1.00	[1.01, 1.02]
Extraversion	1.00	1.00	[1.00, 1.01]	-1.00	1.00	[-0.99, 1.00]	1.01	1.00	[-1.00, 1.01]
Openness	1.00	1.00	[-0.99, 1.00]	-0.99**	1.00	[-0.99, -1.00]	-1.00	1.00	[-0.99, 1.00]
Agreeableness	1.00	1.00	[-1.00, 1.00]	-0.99**	1.00	[-0.99, -0.99]	-1.00	1.00	[-1.00, 1.00]
Conscientiousness	1.00	1.00	[-0.99, 1.00]	-0.99*	1.00	[-0.99, -1.00]	1.00	1.00	[-1.00, 1.01]
Sociodemographic factors									
Sex	-0.95*	1.02	[-0.91, -0.99]	-0.95*	1.03	[-0.90, -1.00]	-1.00	1.00	[-0.96, 1.04]
Age	1.00	1.00	[1.00, 1.00]	-1.00*	1.00	[-1.00, -1.00]	1.00	1.02	[-1.00, 1.00]
Model summary	*F*_18, 4006_ = 39.014, *p* = < .001 adj. *R*^2^ = .145 Durbin-Watson = 1.986 VIF_max_ = 3.271 (Neuroticism)			*F*18, 3993 = 25.125, *p* = < .001 adj. *R*^2^ = .098 Durbin-Watson = 1.992 VIF_max_ = 3.269 (Neuroticism)			*F*18, 3998 = 85.396, *p* = < .001 adj. *R*^2^ = .274 Durbin-Watson = 1.996 VIF_max_ = 3.302 (Neuroticism)		

## Discussion

This large population-based study observed more negative symptom perceptions in individuals with functional disorders with and without co-occuring major health conditions than in those with major health conditions only or healthy individuals. More specifically, individuals with functional disorders judged their symptoms to affect their life and their emotional well-being more and to last longer than the other health condition groups. They expressed less symptom understanding, less treatment control, but higher personal control than those with major health conditions.

These results have three important implications. Firstly, the higher levels of negative cognitive representations and emotional reactions observed in individuals with functional disorders confirm previous research that perceptual, cognitive, and emotion regulation processes may play a more salient role in functional disorders as compared to well-defined physical illness (Henningsen et al., 2018; Okur Güney et al., 2019). Secondly, our results support previous findings from clinical samples that functional disorders in some cases are comorbid with major health conditions (Duffield et al., 2018; Halpin & Ford, 2012). Our results extend the existing evidence by showing that this comorbidity results in more negative symptom perceptions and more negative self-perceived health. Thirdly, more research is needed to investigate the consequences of these more negative symptom perceptions in individuals with functional disorders on relevant outcomes such as symptom burden, symptom course, and individual symptom management.

In terms of correlates of symptom perceptions from the CMS framework, our results indicate that not only the presence of a functional disorder in oneself was associated with symptom perceptions but also functional disorders in family members, albeit to a lesser extent. Interestingly, the presence of a major health condition in the family was not associated with more negative symptom perceptions. These results might indicate that the experience of an illness or symptoms in significant others does not in itself lead to a more negative evaluation of present symptoms but that particularly in functional disorders, learning of illness behavior, and beliefs within families seem to be crucial (Brace et al., 2000; Palermo et al., 2014).

It is of note that the presence of a major health condition, but neither the number of symptoms in the last year, nor the number of symptoms during the last week, was associated with current symptom perceptions in the multivariate regression models. On the one hand, this result might be interpreted in light of former evidence on a weaker association between health states and symptom reports in chronic health conditions (Janssens et al., 2011). On the other hand, the inclusion of functional disorders in the analyses might have erased the impact of symptom reports.

With regard to personality traits, extraversion, openness, and agreeableness were all significantly associated with more favorable symptom perceptions, whereas neuroticism was (to a lesser extent) associated with more negative associations. Notably, conscientiousness was associated with lower personal control. One may speculate that persons with high conscientiousness may need a more controlled environment to feel in control and therefore be prone to appraise less control when experiencing symptoms. Overall, interpretating these results from the perspective of a recent meta-analysis, extraversion, openness, and agreeableness might be regarded as resilience factors in the context of symptom perceptions (Oshio et al., 2018).

In line with the accumulating evidence from other research fields (Anda et al., 2006), multiple experiences of adverse life events were associated with more negative symptom perceptions. Additionally, our results indicate that current symptoms of depression and anxiety as well as perceived stress and coping abilities were psychological correlates of most symptom perceptions. This result was in line with evidence derived from a systematic review on so-called modifiable correlates of symptom perceptions in samples with somatic diseases (Arat et al., 2018) and indicates that these variables might be considered as potential moderators or mediators in future studies.

Taken together, our results support the notion from the perspective of the CSM that a range of biopsychosocial factors are involved in the formation of symptom perceptions (Leventhal et al., 2016), i.e., broadly speaking, that a person's life experience is involved in how the person reacts to and copes with symptoms and illness. Extending on previous evidence, the present study found significant associations between functional disorders in significant others and oneself for the formation of symptom perceptions. Still, the emerging picture is somewhat complex, as it remains challenging to judge which factors might be of particular relevance, given that each B-IPQ subscale displayed an individual pattern of significant biopsychosocial correlates.

From a clinical perspective, screening for functional disorders in individuals with major health conditions may be a valuable approach to identify vulnerable patients that might be at risk for more complex illness trajectories and to personalize the given treatment rationale with psychosocial interventions to challenge symptom perceptions if needed. Derived from the observed associations of symptom perceptions in the present cross-sectional study, these interventions should address present symptoms of depression, anxiety, and current stress and should aim at improving coping skills.

The present study was to the best of our knowledge the first to investigate symptom perceptions and their correlates in a population-based sample. This approach enabled a sufficient sample size and high representativeness. However, the results of the present study should to be interpreted in light of the following limitations. Firstly, the cross-sectional design of the present study prevented us from making any causal/temporal interpretations of our results. Secondly, the participation rate in the DanFunD study was rather low (30%), which is a challenge for all epidemiological studies (Galea & Tracy, 2007). Further, there seemed to be a selection bias, which has also been observed in other epidemiological studies (Keeble et al., 2015), with females and more educated individuals being more likely to participate. Thirdly, the four health condition groups were operationalized through self-report with a predefined list of health conditions. In doing so, some participants may not have indicated a diagnosis of a functional disorder because they disagree with it. Also, other major health conditions not included in this list might have explained some of the perceived symptoms. Fourthly, the present study applied a crude measure of school education. Therefore, the effect of educational level (i.e., vocational training) on the outcome measures has to be investigated in future studies. Fifthly, the B-IPQ uses a single scale approach, which does not allow the determination of internal validity and might be more prone to random measurement error than multi-item scales. Additionally, a scale deviating from the original scale was used and the B-IPQ was answered in terms of symptoms in general, so the item assessing symptom identity was removed. These aspects and large amounts of missing responses on the B-IPQ items decrease the comparability with other studies. Last, further major health conditions or functional disorders and treatment related variables, such as prior illnesses and treatment, symptom duration or severity might be further relevant correlates of symptom perceptions but were not included in the present study.

### Conclusions

Researchers can benefit from the results of the present study with respect to expectable differences in symptom perceptions in healthy individuals and those with functional disorders and major health conditions. Further, the present study identified potential moderators and mediators of symptom perceptions that might be worth further investigation in experimental and treatment studies. Clinicians and health policy makers can benefit from the results in that the present results could inform the future development of preventive interventions in the context of symptom perceptions.

## Data Availability

The datasets generated during and/or analyzed during the current study are available on reasonable request from the DanFunD project leader Thomas Dantoft by email: Thomas.Meinertz.Dantoft@regionh.dk

## Appendix

**Table A.1:** Adjusted Mean Comparisons of B-IPQ Subscales Between the Four Health Condition Groups

B-IPQ subscale	Healthy vs. MHC			Healthy vs. FD			Healthy vs. MHC + FD			MHC vs. FD			MHC vs. MHC + FC			FD vs. MCH + FD		
	M_diff_	95% CI	*d*	M_diff_	95% CI	*d*	M_diff_	[95% CI]	*d*	M_diff_	[95% CI]	*d*	M_diff_	[95% CI]	*d*	M_diff_	[95% CI]	*d*
Consequences	-0.43*	[-0.72, -0.16]	0.20	-1.11*	[-1.31, -0.90]	0.56	-1.20*	[ -1.66, -0.73]	0.61	-0.66*	[-0.98, -0.34 ]	0.32	-0.75*	[-1.27, -0.23 ]	0.35	-0.09	[-0.58, 0.40]	
Timeline	-0.51	[-0.98, -0.03]		-1.51*	[-1.86, -1.15]	0.47	-1.76*	[-2.54, -0.97]	0.58	-1.00*	[-1.55, -0.46]	0.27	-1.25*	[-2.13, -0.37]	0.37	-0.25	[-1.08, 0.58]	
Personal control	-0.22	[-0.63, 0.19]		-0.73*	[-1.03, -0.42]	0.24	-0.46	[-1.14, 0.21]		0.51	[-0.98, -0.04]		-0.24	[-1.01, 0.52]		0.26	[-0.45, 0.98]	
Treatment control	-0.20	[0.64, 0.24]		0.64*	[0.32, 0.97]	0.21	0.41	[-0.32, 1.14]		0.85*	[0.34, 1.35]	0.27	0.62	[-0.20, 1.44]		-0.23	[-1.00, 0.54]	
Symptom concern	-0.20	[-0.52, 0.13]		-0.93*	[-1.17, -0.69]	0.40	-0.98*	[-1.52, -0.44]	0.42	-0.73*	[-1.11, -0.36]	0.31	-0.78*	[-1.40, -0.18]	0.33	-0.05	[0.62, 0.52]	
Coherence	-0.05	[-0.42, 0.32]		-0.47*	[-0.73, -0.20]	0.16	-0.40	[-1.01, 0.21]		-0.42	[-0.84, 0.00]		-0.35	[-1.03, 0.33]		0.07	[-0.57, 0.71]	
Emotional representations	-0.50*	[-0.83, -0.18]	0.17	-1.15*	[-1.38, -0.91]	0.50	-0.53*	[-2.07, -1.00]	0.66	-0.65*	[-1.01, -0.28]	0.45	-1.03*	[-1.63, -0.43]	0.30	-0.39	[-0.95, 0.18]	

*Note.* MHC = majore health condition, i.e. cancer, heart attack and thrombosis or embolism in the brain; FD= functional disorders, i.e. fibromyalgia, chronic fatigue, irritable bowel syndrome, whiplash syndrome, and multiple chemical sensitivity; *d* = Cohen’s d effect size of significant difference; Mdiff = mean difference; 95% CI = 95% confidence interval, Range of B-IPQ subscales = 1-10.

*Significant at Bonferoni corrected *p*-value for multiple comparissons.

## References

[r1] Aalto, A.-M., Aro, A. R., Weinman, J., Heijmans, M., Manderbacka, K., & Elovainio, M. (2006). Sociodemographic, disease status, and illness perceptions predictors of global self-ratings of health and quality of life among those with coronary heart disease – One year follow-up study. Quality of Life Research, 15(8), 1307–1322. 10.1007/s11136-006-0010-3 16826444

[r2] Anda, R. F., Felitti, V. J., Bremner, J. D., Walker, J. D., Whitfield, C., Perry, B. D., Dube, S. R., & Giles, W. H. (2006). The enduring effects of abuse and related adverse experiences in childhood: A convergence of evidence from neurobiology and epidemiology. European Archives of Psychiatry and Clinical Neuroscience, 256(3), 174–186. 10.1007/s00406-005-0624-4 16311898 PMC3232061

[r3] Arat, S., De Cock, D., Moons, P., Vandenberghe, J., & Westhovens, R. (2018). Modifiable correlates of illness perceptions in adults with chronic somatic conditions: A systematic review. Research in Nursing & Health, 41(2), 173–184. 10.1002/nur.21852 29315678

[r4] Bogaerts, K., Van Eylen, L., Li, W., Bresseleers, J., Van Diest, I., De Peuter, S., Stans, L., Decramer, M., & Van den Bergh, O. (2010). Distorted symptom perception in patients with medically unexplained symptoms. Journal of Abnormal Psychology, 119(1), 226–234. 10.1037/a0017780 20141259

[r5] Brace, M. J., Scott Smith, M., McCauley, E., & Sherry, D. D. (2000). Family reinforcement of illness behavior: A comparison of adolescents with chronic fatigue syndrome, juvenile arthritis, and healthy controls. Journal of Developmental and Behavioral Pediatrics, 21(5), 332–339. 10.1097/00004703-200010000-00003 11064960

[r6] Broadbent, E., Petrie, K. J., Main, J., & Weinman, J. (2006). The Brief Illness Perception Questionnaire. Journal of Psychosomatic Research, 60(6), 631–637. 10.1016/j.jpsychores.2005.10.020 16731240

[r7] Broadbent, E., Wilkes, C., Koschwanez, H., Weinman, J., Norton, S., & Petrie, K. J. (2015). A systematic review and meta-analysis of the Brief Illness Perception Questionnaire. Psychology & Health, 30(11), 1361–1385. 10.1080/08870446.2015.1070851 26181764

[r8] Budtz-Lilly, A., Fink, P., Ørnbøl, E., Vestergaard, M., Moth, G., Christensen, K. S., & Rosendal, M. (2015). A new questionnaire to identify bodily distress in primary care: The ‘BDS checklist’. Journal of Psychosomatic Research, 78(6), 536–545. 10.1016/j.jpsychores.2015.03.006 25818346

[r9] Carstensen, T. B. W., Ørnbøl, E., Fink, P., Jørgensen, T., Dantoft, T. M., Madsen, A. L., Buhmann, C. C. B., Eplov, L. F., & Frostholm, L. (2020). Adverse life events in the general population – A validation of the cumulative lifetime adversity measure. European Journal of Psychotraumatology, 11(1), 1717824. 10.1080/20008198.2020.1717824 32128043 PMC7034458

[r10] Christensen, S. S., Frostholm, L., Ørnbøl, E., & Schröder, A. (2015). Changes in illness perceptions mediated the effect of cognitive behavioural therapy in severe functional somatic syndromes. Journal of Psychosomatic Research, 78(4), 363–370. 10.1016/j.jpsychores.2014.12.005 25541119

[r11] Cohen, S., Kamarck, T., & Mermelstein, R. (1983). A global measure of perceived stress. Journal of Health and Social Behavior, 24(4), 385–396. 10.2307/2136404 6668417

[r12] Dantoft, T. M., Ebstrup, J. F., Linneberg, A., Skovbjerg, S., Madsen, A. L., Mehlsen, J., Brinth, L., Eplov, L. F., Carstensen, T. W., Schröder, A., Fink, P. K., Mortensen, E. L., Hansen, T., Pedersen, O., & Jørgensen, T. (2017). Cohort description: The Danish study of functional disorders. Clinical Epidemiology, 9, 127–139. 10.2147/CLEP.S129335 28275316 PMC5333638

[r13] De Gucht, V. (2015). Illness perceptions mediate the relationship between bowel symptom severity and health-related quality of life in IBS patients. Quality of Life Research, 24(8), 1845–1856. 10.1007/s11136-015-0932-8 25663636 PMC4493794

[r14] Dempster, M., Howell, D., & McCorry, N. K. (2015). Illness perceptions and coping in physical health conditions: A meta-analysis. Journal of Psychosomatic Research, 79(6), 506–513. 10.1016/j.jpsychores.2015.10.006 26541550

[r15] Duffield, S. J., Miller, N., Zhao, S., & Goodson, N. J. (2018). Concomitant fibromyalgia complicating chronic inflammatory arthritis: A systematic review and meta-analysis. Rheumatology, 57(8), 1453–1460. 10.1093/rheumatology/key112 29788461 PMC6055651

[r16] Fink, P., Ørnbøl, E., Huyse, F. J., De Jonge, P., Lobo, A., Herzog, T., Slaets, J., Arolt, V., Cardoso, G., Rigatelli, M., & Hansen, M. S. (2004). A brief diagnostic screening instrument for mental disturbances in general medical wards. Journal of Psychosomatic Research, 57(1), 17–24. 10.1016/S0022-3999(03)00374-X 15256291

[r17] Frostholm, L., Ørnbøl, E., Christensen, K. S., Toft, T., Olesen, F., Weinman, J., & Fink, P. (2007). Do illness perceptions predict health outcomes in primary care patients? A 2-year follow-up study. Journal of Psychosomatic Research, 62(2), 129–138. 10.1016/j.jpsychores.2006.09.003 17270570

[r18] Galea, S., & Tracy, M. (2007). Participation rates in epidemiologic studies. Annals of Epidemiology, 17(9), 643–653. 10.1016/j.annepidem.2007.03.013 17553702

[r19] Goetzmann, L., Scheuer, E., Naef, R., Klaghofer, R., Russi, E. W., Buddeberg, C., & Boehler, A. (2005). Personality, illness perceptions, and lung function (FEV_1_) in 50 patients after lung transplantation. Psycho-Social Medicine, 2, 1–6. http://www.egms.de/en/journals/psm/2005-2/psm000015.shtml 19742058 PMC2736488

[r20] Hagger, M. S., Koch, S., Chatzisarantis, N. L. D., & Orbell, S. (2017). The common sense model of self-regulation: Meta-analysis and test of a process model. Psychological Bulletin, 143(11), 1117–1154. 10.1037/bul0000118 28805401

[r21] Halpin, S. J., & Ford, A. C. (2012). Prevalence of symptoms meeting criteria for irritable bowel syndrome in inflammatory bowel disease: Systematic review and meta-analysis. The American Journal of Gastroenterology, 107(10), 1474–1482. 10.1038/ajg.2012.260 22929759

[r22] Henningsen, P., Zipfel, S., Sattel, H., & Creed, F. (2018). Management of functional somatic syndromes and bodily distress. Psychotherapy and Psychosomatics, 87(1), 12–31. 10.1159/000484413 29306954

[r23] Hinz, A., Ernst, J., Glaesmer, H., Brähler, E., Rauscher, F. G., Petrowski, K., & Kocalevent, R.-D. (2017). Frequency of somatic symptoms in the general population: Normative values for the Patient Health Questionnaire-15 (PHQ-15). Journal of Psychosomatic Research, 96, 27–31. 10.1016/j.jpsychores.2016.12.017 28545789

[r24] Horwood, S., & Anglim, J. (2017). A critical analysis of the assumptions of Type D personality: Comparing prediction of health-related variables with the Five Factor Model. Personality and Individual Differences, 117, 172–176. 10.1016/j.paid.2017.06.001

[r25] Janssens, T., Verleden, G., De Peuter, S., Petersen, S., & Van den Bergh, O. (2011). The influence of fear of symptoms and perceived control on asthma symptom perception. Journal of Psychosomatic Research, 71(3), 154–159. 10.1016/j.jpsychores.2011.04.005 21843750

[r26] Joustra, M. L., Janssens, K. A., Bultmann, U., & Rosmalen, J. G. (2015). Functional limitations in functional somatic syndromes and well-defined medical diseases: Results from the general population cohort LifeLines. Journal of Psychosomatic Research, 79(2), 94–99. 10.1016/j.jpsychores.2015.05.004 26026696

[r27] Keeble, C., Baxter, P. D., Barber, S., & Law, G. R. (2015). Participation rates in epidemiology studies and surveys: A review 2007–2015. Internet Journal of Epidemiology, 14(1). 10.5580/IJE.34897

[r28] Körner, A., Geyer, M., & Brähler, E. (2002). Das NEO-Fünf-Faktoren Inventar (NEO-FFI). Diagnostica, 48(1), 19–27. 10.1026//0012-1924.48.1.19

[r29] Leventhal, H., Phillips, L. A., & Burns, E. (2016). The Common-Sense Model of Self-Regulation (CSM): A dynamic framework for understanding illness self-management. Journal of Behavioral Medicine, 39(6), 935–946. 10.1007/s10865-016-9782-2 27515801

[r30] Luszczynska, A., Scholz, U., & Schwarzer, R. (2005). The general self-efficacy scale: Multicultural validation studies. The Journal of Psychology, 139(5), 439–457. 10.3200/JRLP.139.5.439-457 16285214

[r31] Mols, F., Denollet, J., Kaptein, A. A., Reemst, P. H., & Thong, M. S. (2012). The association between Type D personality and illness perceptions in colorectal cancer survivors: A study from the population-based PROFILES registry. Journal of Psychosomatic Research, 73(3), 232–239. 10.1016/j.jpsychores.2012.07.004 22850265

[r32] O’Donovan, C. E., Painter, L., Löwe, B., Robinson, H., & Broadbent, E. (2016). The impact of illness perceptions and disease severity on quality of life in congenital heart disease. Cardiology in the Young, 26(1), 100–109. 10.1017/S1047951114002728 25599956

[r33] Okur Güney, Z. E., Sattel, H., Witthöft, M., & Henningsen, P. (2019). Emotion regulation in patients with somatic symptom and related disorders: A systematic review. PLoS One, 14(6), e0217277. 10.1371/journal.pone.0217277 31173599 PMC6555516

[r34] Olsen, L. R., Mortensen, E. L., & Bech, P. (2004). The SCL-90 and SCL-90R versions validated by item response models in a Danish community sample. Acta Psychiatrica Scandinavica, 110(3), 225–229. 10.1111/j.1600-0447.2004.00399.x 15283743

[r35] Oshio, A., Taku, K., Hirano, M., & Saeed, G. (2018). Resilience and Big Five personality traits: A meta-analysis. Personality and Individual Differences, 127(1), 54–60. 10.1016/j.paid.2018.01.048

[r36] Palermo, T. M., Valrie, C. R., & Karlson, C. W. (2014). Family and parent influences on pediatric chronic pain: A developmental perspective. The American Psychologist, 69(2), 142–152. 10.1037/a0035216 24547800 PMC4056332

[r37] Petersen, S., van den Berg, R. A., Janssens, T., & Van den Bergh, O. (2011). Illness and symptom perception: A theoretical approach towards an integrative measurement model. Clinical Psychology Review, 31(3), 428–439. 10.1016/j.cpr.2010.11.002 21146271

[r38] Petrie, K. J., Cameron, L. D., Ellis, C. J., Buick, D., & Weinman, J. (2002). Changing illness perceptions after myocardial infarction: An early intervention randomized controlled trial. Psychosomatic Medicine, 64(4), 580–586. 10.1097/00006842-200207000-00007 12140347

[r39] Princip, M., Gattlen, C., Meister-Langraf, R. E., Schnyder, U., Znoj, H., Barth, J., Schmid, J. P., & von Känel, R. (2018). The role of illness perception and its association with posttraumatic stress at 3 months following acute myocardial infarction. Frontiers in Psychology, 9, 941. 10.3389/fpsyg.2018.00941 29930529 PMC5999791

[r40] Rassart, J., Luyckx, K., Klimstra, T. A., Moons, P., Groven, C., & Weets, I. (2014). Personality and illness adaptation in adults with type 1 diabetes: The intervening role of illness coping and perceptions. Journal of Clinical Psychology in Medical Settings, 21(1), 41–55. 10.1007/s10880-014-9387-2 24496583

[r41] Sheldrick, R., Tarrier, N., Berry, E., & Kincey, J. (2006). Post-traumatic stress disorder and illness perceptions over time following myocardial infarction and subarachnoid haemorrhage. British Journal of Health Psychology, 11(3), 387–400. 10.1348/135910705X71434 16870051

[r42] Tiemensma, J., Gaab, E., Voorhaar, M., Asijee, G., & Kaptein, A. A. (2016). Illness perceptions and coping determine quality of life in COPD patients. International Journal of Chronic Obstructive Pulmonary Disease, 11(1), 2001–2007. 10.2147/COPD.S109227 27601893 PMC5003510

[r43] Timmers, L., Thong, M., Dekker, F. W., Boeschoten, E. W., Heijmans, M., Rijken, M., Weinman, J., Kaptein, A., & the Netherlands Cooperative Study on the Adequacy of Dialysis Study Group. (2008). Illness perceptions in dialysis patients and their association with quality of life. Psychology & Health, 23(6), 679–690. 10.1080/14768320701246535 25160810

[r44] Tribbick, D., Salzberg, M., Connell, W., Macrae, F., Kamm, M., Bates, G., Cunningham, G., Austin, D., & Knowles, S. (2017). Differences across illness perceptions in inflammatory bowel disease and their relationships to psychological distress and quality of life. Gastroenterology Nursing, 40(4), 291–299. 10.1097/SGA.0000000000000225 28746114

[r45] Van den Bergh, O., Witthoft, M., Petersen, S., & Brown, R. J. (2017). Symptoms and the body: Taking the inferential leap. Neuroscience and Biobehavioral Reviews*,* 74(Pt A), 185-203. 10.1016/j.neubiorev.2017.01.015 28108416

[r46] van Erp, S. J. H., Brakenhoff, L. K. M. P., Vollmann, M., van der Heijde, D., Veenendaal, R. A., Fidder, H. H., Hommes, D. W., Kaptein, A. A., van der Meulen-de Jong, A. E., & Scharloo, M. (2017). Illness perceptions and outcomes in patients with inflammatory bowel disease: Is coping a mediator? International Journal of Behavioral Medicine, 24(2), 205–214. 10.1007/s12529-016-9599-y 27757843

[r47] Ware, J., Jr., Kosinski, M., & Keller, S. D. (1996). A 12-Item Short-Form Health Survey: Construction of scales and preliminary tests of reliability and validity. Medical Care, 34(3), 220–233. 10.1097/00005650-199603000-00003 8628042

[r48] Whitaker, K. L., Scott, S. E., & Wardle, J. (2015). Applying symptom appraisal models to understand sociodemographic differences in responses to possible cancer symptoms: A research agenda. British Journal of Cancer, 112(Suppl 1), S27–S34. 10.1038/bjc.2015.39 25734385 PMC4385973

[r49] Xiong, N. N., Wei, J., Ke, M. Y., Hong, X., Li, T., Zhu, L. M., Sha, Y., Jiang, J., & Fischer, F. (2018). Illness perception of patients with functional gastrointestinal disorders. Frontiers in Psychiatry, 9, 122. 10.3389/fpsyt.2018.00122 29706904 PMC5906533

